# Supplementary material for: Accuracy of heart rate measurement using AirPods Pro 3 during graded treadmill exercise: A laboratory-based validation study
Source: PLOS Digit Health. 2026 Apr 30;5(4):e0001373. doi: 10.1371/journal.pdig.0001373 (PMC13132241; doi:10.1371/journal.pdig.0001373)
Supplement: Attachment — Submitted filename: PDIG_Reviewer_Comments_250226.docx [file pdig.0001373.s002.docx]

**Additional Editor Comments (if provided):**

This is a great project and interesting data and as the use of wearables from consumer to medical grade devices will increase globally over coming years, and more applications are integrated into such devices, these studies are very important. Congratulations to the team on this research.

RESPONSE: Thank you for your kind appraisal of our work – and we wholeheartedly agree that these kinds of studies are needed to keep up with the commercial ecosystem.
Minor revisions:

- At 476 Data Processing – further expand on rationale for the AirPods pro heart rate values analysis to further explore rationale if aim is to process as raw data so to speak with no filtration for AirPods.

RESPONSE: We have expanded the Data Processing section to clarify that AirPods heart-rate values were analysed exactly as provided to end users in order to preserve ecological validity and avoid artificially improving agreement through investigator-applied filtering. This approach aligns with INTERLIVE recommendations to evaluate wearable devices at the user-level output.

Methodological Transparency -

- Is there any further information regarding a literature review or specifics around key word or search strategy such as systematically searching PubMed, MEDLINE, Embase, Web of Science, Global Health, Cochrane Library, APA PsycINFO, and APA PsycArticles etc for studies published?

RESPONSE: This study is an original device validation study and did not aim to conduct a systematic review of the literature. The background references were selected through targeted narrative review to contextualise the study within existing evidence on wearable heart-rate validation, with particular emphasis on recent systematic reviews, meta-analyses, and key methodological guidance (e.g., INTERLIVE recommendations). 

Formatting and Layout Clarification –

- Reformatting the layout of the research article to ensure that Search Strategy relating to any literature review and references, Quality Assessment and Materials and Methods is outlined prior to the Discussion and Conclusion statement to align with publishing formats.

RESPONSE: As per previous comment, this manuscript reports an original laboratory-based device validation study and does not include a systematic review component. Accordingly, no formal search strategy or study quality assessment was conducted. The manuscript is structured according to the standard format for primary research articles, with detailed methodological information provided in the Materials and Methods section. We have reviewed the manuscript structure to ensure alignment with PLOS Digital Health formatting requirements and confirm that the current organisation reflects the appropriate format for an experimental validation study.

- Additionally ensure referencing formatting is in line with PLOS Digital Health guidelines.

RESPONSE: Confirmed.

Discussion Considerations (pending word limit considerations):

- Consider further additional commentary regarding limitations of cohort size, demographic and in particular Fitzpatrick skin types to ensure future research is considered regarding cultural and genetic factors that may impact heart rate detection.

- As the potential users continue to grow, wearables will have more sociological and cultural impact in the future. Consideration to expand on the current comments regarding cohort and future studies may be useful to support exploration of technical development, health infrastructure, digital connectivity and digital and technology health equity resources. In relation to future research, the most vulnerable or most affected populations who may have health and technology disparity in access would need to be considered to ensure broad generalisations are not made regarding the application of this technology.

- Furthermore considerations regarding interpretation of clinical bias for future research study designs, in relation to the data regarding age predicted maximal heart rate and careful interpretation to assess what this may be for different cultural, and patient cohorts - this may affect how the data is interpreted and therefore how it is used by consumers or health professionals.
For example in Indigenous populations in other countries, and countries such as Australia with higher rates of cardiovascular and rheumatic heart disease there is a higher risk of cardiovascular disease including arrhythmias, or if there are considerations regarding genetically predisposed cohorts with cardiac conditions that future studies may need to be mindful of regarding what is considered "normal" for that particular cohort.

Additionally, in the discussion section for future research considerations regarding the applicability and results of the use of the technology in rural and remote health care settings, and in particular technology and health literacy to ensure these results are not widely applied without further research and highlighting challenges for populations in rural and remote communities regarding connectivity and digital satellite access may impact technology use and results, in addition to the reliability of the data and its use.

RESPONSE: We have expanded the Discussion to provide a more detailed consideration of population diversity and contextual factors that may influence the generalisability and real-world implications of these findings. The revised text now explicitly addresses the under-representation of darker Fitzpatrick skin types, the potential for differential performance in populations with cardiovascular disease or population-specific physiological characteristics, and the limitations of using age-predicted maximal heart rate across diverse demographic groups. We have also added discussion of broader digital health considerations, including access to smartphones, connectivity, digital literacy, and potential challenges for implementation in rural, remote, and low-resource settings. These additions emphasise the need for future research in more diverse populations and real-world contexts and clarify that findings should not be overgeneralised beyond the study population. (Discussion, page 16-17).

[Note: HTML markup is below. Please do not edit.]

**Reviewers' Comments:**

[NOTE: If reviewer comments were submitted as an attachment file, they will be attached to this email and accessible via the submission site. Please log into your account, locate the manuscript record, and check for the action link "View Attachments". If this link does not appear, there are no attachment files.]

**Figure resubmission:**

While revising your submission, we strongly recommend that you use PLOS’s NAAS tool (https://ngplosjournals.pagemajik.ai/artanalysis) to test your figure files. NAAS can convert your figure files to the TIFF file type and meet basic requirements (such as print size, resolution), or provide you with a report on issues that do not meet our requirements and that NAAS cannot fix.

After uploading your figures to PLOS’s NAAS tool - https://ngplosjournals.pagemajik.ai/artanalysis, NAAS will process the files provided and display the results in the "Uploaded Files" section of the page as the processing is complete. If the uploaded figures meet our requirements (or NAAS is able to fix the files to meet our requirements), the figure will be marked as "fixed" above. If NAAS is unable to fix the files, a red "failed" label will appear above. When NAAS has confirmed that the figure files meet our requirements, please download the file via the download option, and include these NAAS processed figure files when submitting your revised manuscript.

**Reproducibility:**

To enhance the reproducibility of your results, we recommend that authors of applicable studies deposit laboratory protocols in protocols.io, where a protocol can be assigned its own identifier (DOI) such that it can be cited independently in the future. Additionally, PLOS ONE offers an option to publish peer-reviewed clinical study protocols. Read more information on sharing protocols at https://plos.org/protocols?utm_medium=editorial-email&utm_source=authorletters&utm_campaign=protocols
